# A Novel *fry1* Allele Reveals the Existence of a Mutant Phenotype Unrelated to 5′->3′ Exoribonuclease (XRN) Activities in *Arabidopsis thaliana* Roots

**DOI:** 10.1371/journal.pone.0016724

**Published:** 2011-02-03

**Authors:** Judith Hirsch, Julie Misson, Peter A. Crisp, Pascale David, Vincent Bayle, Gonzalo M. Estavillo, Hélène Javot, Serge Chiarenza, Allison C. Mallory, Alexis Maizel, Marie Declerck, Barry J. Pogson, Hervé Vaucheret, Martin Crespi, Thierry Desnos, Marie-Christine Thibaud, Laurent Nussaume, Elena Marin

**Affiliations:** 1 CEA, DSV IBEB, Laboratoire de Biologie du Développement des Plantes, UMR 6191 CNRS, CEA, Aix-Marseille II, Saint-Paul-lez-Durance, France; 2 ARC Centre of Excellence in Plant Energy Biology, Research School of Biology, Australian National University, Canberra, Australian Capital Territory, Australia; 3 Institut Jean-Pierre Bourgin, UMR 1318, INRA, Versailles, France; 4 Department of Stem Cell Biology, University of Heidelberg, Heidelberg, Germany; 5 Institut des Sciences du Végétal, CNRS, Gif sur Yvette, France; University of Melbourne, Australia

## Abstract

**Background:**

Mutations in the *FRY1/SAL1* Arabidopsis locus are highly pleiotropic, affecting drought tolerance, leaf shape and root growth. *FRY1* encodes a nucleotide phosphatase that *in vitro* has inositol polyphosphate 1-phosphatase and 3′,(2′),5′-bisphosphate nucleotide phosphatase activities. It is not clear which activity mediates each of the diverse biological functions of FRY1 *in planta*.

**Principal Findings:**

A *fry1* mutant was identified in a genetic screen for Arabidopsis mutants deregulated in the expression of Pi High affinity Transporter 1;4 (*PHT1;4*). Histological analysis revealed that, in roots, *FRY1* expression was restricted to the stele and meristems. The *fry1* mutant displayed an altered root architecture phenotype and an increased drought tolerance. All of the phenotypes analyzed were complemented with the *AHL* gene encoding a protein that converts 3′-polyadenosine 5′-phosphate (PAP) into AMP and Pi. PAP is known to inhibit exoribonucleases (XRN) *in vitro*. Accordingly, an *xrn* triple mutant with mutations in all three XRNs shared the *fry1* drought tolerance and root architecture phenotypes. Interestingly these two traits were also complemented by grafting, revealing that drought tolerance was primarily conferred by the rosette and that the root architecture can be complemented by long-distance regulation derived from leaves. By contrast, *PHT1* expression was not altered in *xrn* mutants or in grafting experiments. Thus, *PHT1* up-regulation probably resulted from a local depletion of Pi in the *fry1* stele. This hypothesis is supported by the identification of other genes modulated by Pi deficiency in the stele, which are found induced in a *fry1* background.

**Conclusions/Significance:**

Our results indicate that the 3′,(2′),5′-bisphosphate nucleotide phosphatase activity of FRY1 is involved in long-distance as well as local regulatory activities in roots. The local up-regulation of *PHT1* genes transcription in roots likely results from local depletion of Pi and is independent of the XRNs.

## Introduction

In the last ten years, a variety of independent genetic screens have identified defects in the enzyme FIERY1/SAL1 (FRY1). The first *fry1* mutants were identified in a genetic screen based on the deregulation of an ABA reporter gene [1]. FRY1 was described as a repressor of ABA-mediated stress signal transduction, as the corresponding mutant presented an increased sensitivity to cold, salt and drought stresses [1]. FRY1 seems to act as a negative regulator of both ABA-independent and ABA-dependent stress response pathway ([2], Estavillo and Pogson, personal communication) and is involved in leaf venation patterning [3]. Independent screens also identified *fry1* alleles affecting the regulation of photo-morphogenic processes, including hypocotyl elongation and flowering time [4] and lateral root initiation [5].

Such a diversity of phenotypes could be explained by the complexity of FRY1 activity. FRY1 was originally identified as a bifunctional enzyme presenting both an inositol polyphosphate 1-phosphatase activity that hydrolyses inositol 1,4,5-trisphosphate (IP3) *in vitro*, complementing a salt sensitive yeast strain [6], and a highly specific 3′,(2′),5′-bisphosphate nucleotide phosphatase activity converting PAP (3′-polyadenosine 5′-phosphate) into AMP and phosphate (Pi) [6], [7]. This latter activity was predicted to negatively impact the amount of PAP available in the cell. Indeed, in a separate paper that focuses on chloroplast to nuclear signaling in leaves, several authors from the current study shown that PAP content and not inositol phosphates are regulated by FRY1/SAL1 (Estavillo and Pogson, personal communication). *In vitro*, PAP suppresses the activity of the yeast 5′->3′ exoribonucleases Rat1 and Xrn1 [8]. Thus, the accumulation of PAP in loss-of-function *fry1* mutants could inhibit the three Rat1 Arabidopsis orthologs XRN2, XRN3 and XRN4, which are all RNA silencing-suppressors [9]. Indeed, some phenotypes of *xrn3*, *xrn2 xrn3* and *xrn4* mutants mimic some *fry1* traits such as an altered leaf shape, hypocotyl length and reduction of lateral root initiation [4], [5], [9]. Nevertheless, the contribution of the roots to the reported *alx8* and *fry1-1* drought tolerance [2] and the role of XRNs in root morphology and drought tolerance have not been analyzed.

Using a reporter gene strategy to identify mutations deregulating the expression of the high affinity phosphate transporter *PHT1;4* [10], we identified a novel allele of *fry1*. In addition to root deregulation of the gene reporter, the mutant exhibited strong root architecture defects and a drought resistance phenotype. Through physiological approaches, grafting experiments and mutant analysis, we show that FRY1 plays a role in long distance signaling to roots through its proposed impact on XRN activities in leaves. In contrast, we reveal a new role for FRY1 in the local regulation of phosphate starvation response genes likely linked to a local depletion of Pi in the root stele.

## Results and Discussion

### Identification of a mutant deregulating *PHT1;4::GUS* expression and root development

When driven by the promoter of the high affinity phosphate transporter gene *PHT1;4*, the GUS reporter gene is induced by phosphate starvation and primarily expressed in the Arabidopsis root. We screened seedlings for the deregulation of this root-expressed reporter gene, in an EMS-derived population of a transgenic line. In our screening conditions (i.e. on phosphate-rich medium) the expression of this reporter marker was not detectable in roots of the parental line [10].

**Figure 1:**
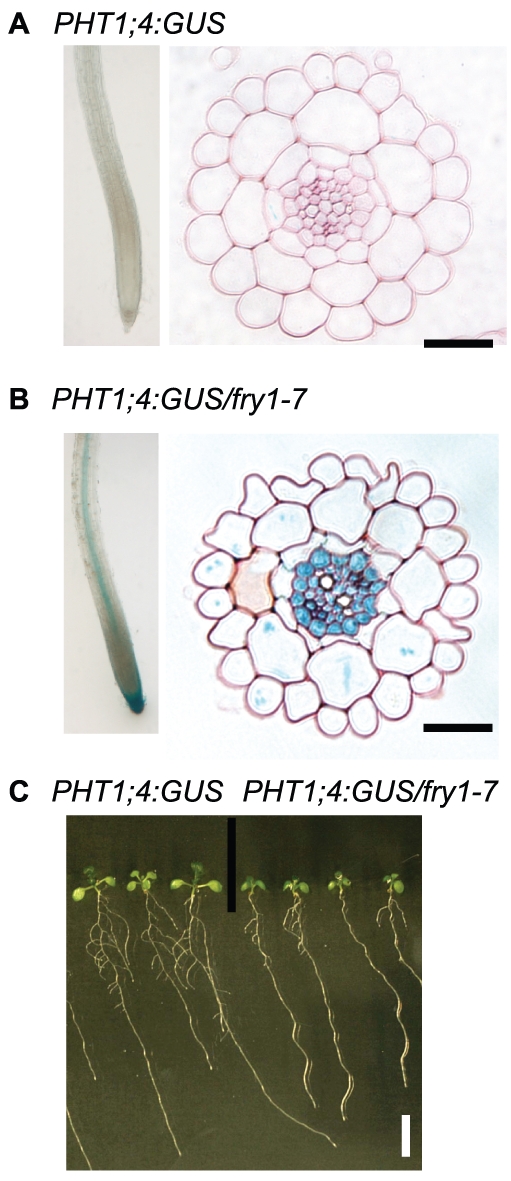
Phenotype of the *fry1-7* mutant. (A-B) Root cross section of a ten day-old *PHT1;4:GUS* parental line (A) and the *PHT1;4:GUS/fry1-7* mutant (B) after GUS staining. Scale bars, 30 µm. (C) Ten day-old plantlets of the *PHT1;4:GUS* parental line and the *PHT1;4:GUS/fry1-7* mutant, note the reduced root system in the mutant. Scale bar, 10 mm.

Ten day-old seedlings from each of the 1400 M2 families were stained and roots were screened for seedlings with detectable GUS expression [11]. We identified a recessive mutant (*fry1-7*, see below) that constitutively expressed the GUS reporter gene in the central cylinder and the pericycle of the root and in primary root meristems (Fig. 1A, B). This mutant also displayed shorter primary and lateral roots (Fig. 1C).

### Map-based cloning identifies a new allele of *fry1*

**Figure 2:**
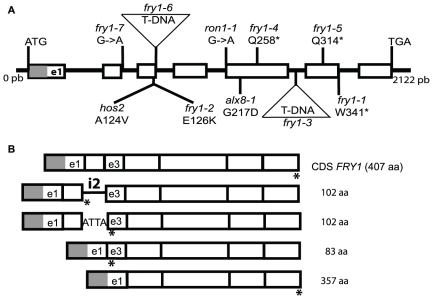
Schematic of the mutant *fry1* alleles. (A) *FRY1* gene structure and position of *fry1* mutations. White boxes represent the exons, the horizontal lines represent the introns and the UTRs. In the first exon (e1) the grey box corresponds to the plastid transit peptide (54 amino acids long) predicted in the TAIR database. Positions of the T-DNA insertion in the *fry1-3* and *fry1-6* mutants alleles are indicated by triangles. The nature of the untagged *fry1* alleles is indicated: Lines indicate point mutations, numbers show the position of amino acids, asterisks indicate stop codons. (B) CDS of the *FRY1* locus and structure of different splice variants identified in the *fry1-7* mutant. In one of the splice variants, the second intron (i2) has been conserved due to the point mutation in *fry1-7*. The 3^rd^ exon is marked (e3) to clarify the interpretation of the figure. The protein length indicated for each splice variant includes the 54 amino acids of the transit peptide. Asterisks indicate stop codons.

The mutation was mapped on chromosome 5 between microsatellite markers 5.74 and 5.80, which define an approximately 110 kb interval containing 29 genes. A transcriptomic analysis showed that a transcript corresponding to At5g63980 (*FRY1/HOS2/SAL1*) was down-regulated in the mutant to 30% of the level detected in the *PHT1;4:GUS* parental line. Sequencing of the corresponding locus in the mutant line revealed a point mutation (G to A exchange) at nucleotide position 559 in the donor site of the second intron of the *FRY1* genomic sequence (Fig. 2A). This mutation altered the splicing of *FRY1* transcripts, as confirmed by the cloning and sequencing of four *FRY1* splice variants in the mutant (Fig. 2B). All splice variants encoded truncated forms of the FRY1 protein, suggesting that this mutant, referred to as *fry1-7*, is a loss-of-function allele. Expression of the *FRY1* cDNA under the control of the *35S* promoter in the *fry1-7* line complemented the root phenotype (Fig. S1A), confirming that the mutation in *fry1* was responsible for the root defect. Importantly, the expression of the *GUS* reporter was also complemented in *PHT1;4:GUS/fry1-7/35S::FRY1* lines (data not shown) and was indistinguishable from the original *PHT1;4:GUS* line. An allelism test between *fry1-7* and the T-DNA insertion allele *fry1-6* (Fig. 2A; [9]) further confirmed that *FRY1* was the causal gene (data not shown).

Together with the root developmental defects, *fry1-7* mutants displayed the aerial growth and developmental defects previously described for other *fry1* alleles, including *fry1-6* [2], [4], [9]. Young rosette leaves were crinkly and presented rounded leaf margins and shorter petioles (Fig. S1B), whereas older leaves were serrated. In addition, when transferred to soil, the mutant was more tolerant to drought stress than the wild type control (see below) and displayed a general delay in growth (Fig. S1C) and flowering time (data not shown).

### *fry1* stimulates the transcription of several genes induced by Pi starvation in the stele

**Figure 3:**
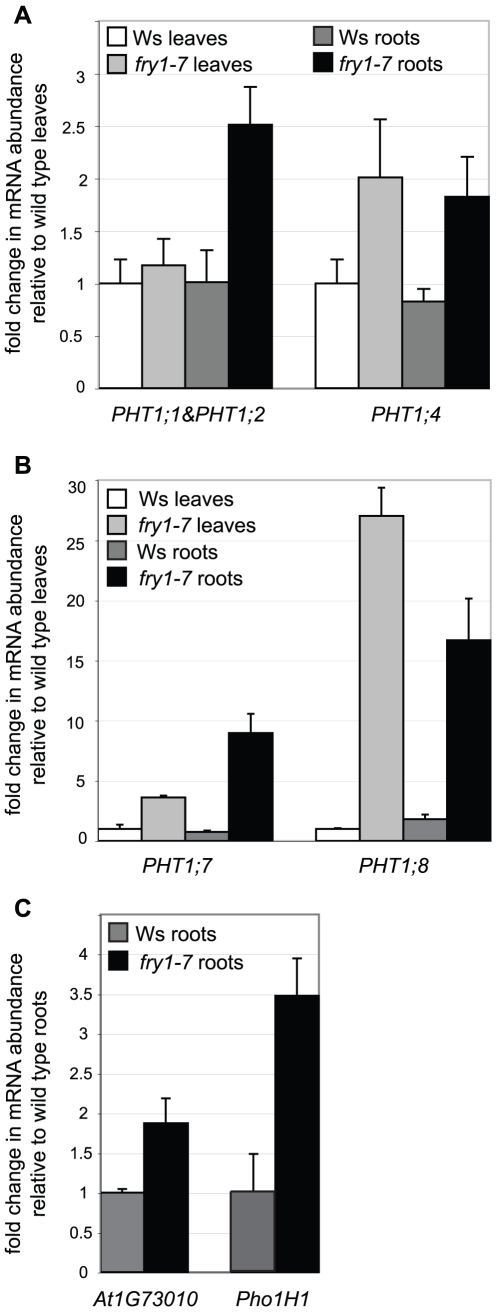
Expression of phosphate induced genes in leaves and roots of the *fry1-7* mutant. (A) Quantitative real time PCR of the *PHT1;1* *&PHT1;2* and *PHT1;4* transcripts in *fry1-7* and Ws plantlets. (B) Quantitative real time PCR on the *PHT1;7* and *PHT1;8* loci in *fry1-7* and Ws plantlets. (C) Quantitative real time PCR on the *At1G73010* and *Pho1H1* loci in *fry1-7* and Ws roots. Biological triplicates were performed and all samples were analyzed with technical triplicates. White bars correspond to Ws leaves, pale grey bars to *fry1-7* leaves, dark grey bars to Ws roots and black bars to *fry1-7* roots. Standard deviations are shown.

To test whether the GUS expression in *PHT1;4:GUS/fry1* was due to the upregulation of the endogenous *PHT1*,*4* gene or specific to the T-DNA reporter construct inserted in *PHT1;4*, we generated a *fry1-7* line devoid of any T-DNA insertion by performing a series of back-crosses. We then measured the *PHT1;4* expression level in this line by qRT-PCR. In both leaves and roots, we observed an increase in *PHT1;4* transcript levels in the *fry1-7* single mutant as compared to the Ws control (Fig. 3A). The induction of *PHT1;4* was also detected in the *fry1-6* allele (Fig. S3), which confirms that the expression of the *PHT1;4:GUS* transgene in the *PHT1;4:GUS/fry1-7* mutant reflects the activation of the endogenous *PHT1;4* gene. Thus, in high phosphate conditions, *fry1* mutants show a constitutive induction of *PHT1;4* in the central cylinder of the root.

We tested whether the *fry1* mutation stimulates the expression of other genes related to *PHT1-4*. This phosphate transporter belongs to a multigenic family (the *PHT1* gene family) that exhibits a tight co-regulation (in particular during Pi deficiency [12]). We found that *PHT1;1* and *PHT1;2* (revealed by a common pair of primers), *PHT1;7* and *PHT1;8* transcripts were also induced in *fry1-7* (Fig. 3A, B) as compared to the wild type control.

In order to assay if genes modulated by Pi starvation distinct from *PHT1* family could also be affected by *fry1* mutation, we tested two other markers associated with Pi deficiency in the stele: *Pho1H1* [13] and the At1G73010 phosphatase (Fig. S2). Both genes were found significantly induced in roots of the *fry1* background (Fig. 3C and Fig. S4C, D). Analyses revealed an absence of obvious alterations in Pi content, uptake or transport capacity of the *fry1* mutant (data not shown). Nevertheless, the levels of gene induction measured here by qRT-PCR are substantially lower than those observed during phosphate starvation [12], [14]. This suggested that the reduction of Pi level is probably limited. In addition, such variation should be restricted to the root stele and masked by the accumulation of vacuolar Pi in external root cell layers such as cortex and epidermis. It is therefore not surprising that such specific Pi discrepancies could not be detected by available techniques and only visualized by the use of sensitive reporter genes or by PCR techniques.

### Altered root architecture in *fry1* mutants is due to reduced meristem activity in the PR and to an LR initiation defect

**Figure 4:**
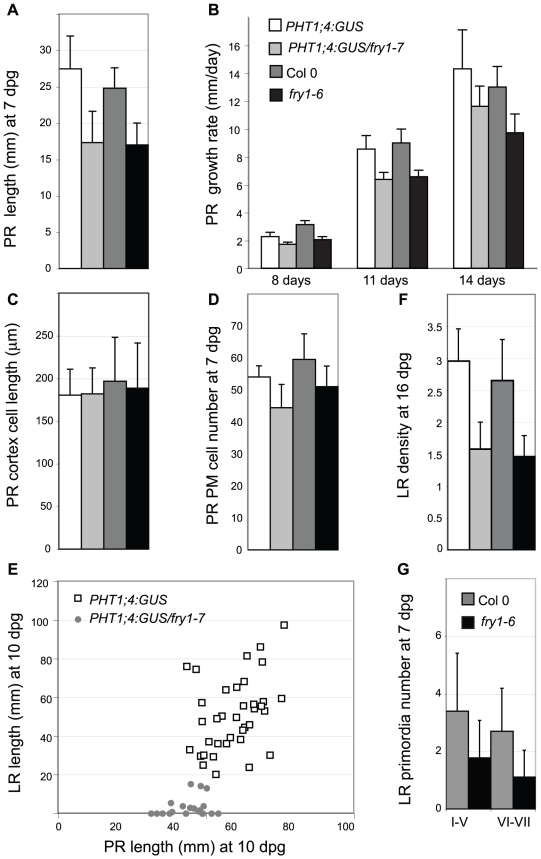
Root architecture of the *fry1* mutants. (A) Primary root length at 7 days post germination (dpg). (B) Growth rate of the primary root at 8, 11 and 14 dpg on MS/10 medium. (C) Primary root cortical cell length. (D) Primary root proximal meristem (PM) cell number at 7 dpg. (E) Diagram plotting total lateral root length *vs* primary root length of the *PHT1;4:GUS* line (white squares) and *PHT1;4:GUS*/*fry1-7* mutant (grey circles). 18 to 30 plants were measured per genotype, 10 dpg. (F) LR density of *fry1* (number of LR per mm PR) at 16 dpg. (G) Number of LR primordia at early stages (I-V) and late stages (VI-VII), 7 dpg. The wild type and the mutant in A, B, D and G are significantly different (P<0.01) (Student's t-test). For A–D and F the white bars correspond to the *PHT1;4:GUS* parental line, the *PHT1;4:GUS*/*fry1-7* mutant appears in pale grey, Col in dark grey and the *fry1-6* mutant in black, as detailed in panel B. For all the analyses, at least three independent experiments gave similar results. Standard deviations are shown.

Alteration of *fry1* root architecture has been recently reported [5], but the description of the root phenotype was limited to lateral root initiation. Our analysis indicated that the root system of the *fry1-7* mutant is reduced compared to the parental control line both at the primary root (PR) and the lateral root (LR) levels. Seven days post germination (dpg), the *fry1-7* mutant primary root was 37% shorter than its parental line, and the *fry1-6* primary root was 32% shorter than the Col PR (Fig. 4A). Quantification of PR growth rate during *in vitro* development in both the *fry1-7* and the *fry1-6* mutant alleles revealed a statistically significant difference in growth rate when compared with controls (determined by Student's t test, P<0.01), which likely explains the growth delay observed in the mutant (Fig. 4B).

Reduced root growth can result from a defect in cell elongation and/or from a decrease in meristem activity. Measuring cortical cell length did not reveal any differences between *fry1* alleles and wild type controls (Fig. 4C). However, PR cell number in the proximal meristem (PM) at 7dpg [15] was mildly reduced, although statistically significant, in the *fry1-6* and *fry1-7* mutants when compared to the wild type PM size (Fig. 4D). These results show that the modified PR growth observed in *fry1* is due to a defect in maintenance and/or activity of the root apical meristem.

The *fry1* mutation also reduced the LR length (Fig. 4E), the LR density (Fig. 4F) and the LR primordia number (Fig. 4G). Thus, it is likely that the altered root architecture of *fry1* mutants is not only due to a delay in growth. Interestingly, LR cortical cell length and PM cell number were comparable among *fry1-7* and *fry1-6* alleles and the corresponding wild type plants when measured at 14 dpg (data not shown), suggesting that an independent factor limits LR initiation or progression. Auxin is a good candidate for such a factor as the *fry1* mutant has reduced auxin sensitivity at the level of LR initiation [5]. Nevertheless, this auxin response defect could not explain all *fry1* root traits as the *fry1* PR exhibited auxin sensitivity similar to wild type (data not shown).

### The 3′,(2′),5′-bisphosphate nucleotide phosphatase activity complements the root mutant phenotype of *fry1* as well as the *PHT1;4:GUS* induction

FRY1 is a bifunctional enzyme whereas *AHL* (Arabidopsis HAL2-like, At5g54390) is a *FRY1* paralog encoding a protein with only the 3′,(2′),5′-bisphosphate nucleotide phosphatase activity *in vitro* [7]. In order to test whether the 3′,(2′),5′-bisphosphate nucleotide phosphatase activity is sufficient to recover wild type root and *PHT1;4* induction level, we used the *AHL* gene harboring only this activity (i.e. not the inositol polyphosphate 1-phosphatase activity). Overexpression of *AHL* complemented the root phenotype of the *fry1* mutant (Fig. S1D), indicating that the altered root growth of *fry1* mutants is likely to be due to the lack of the FRY1 3′,(2′),5′-bisphosphate nucleotide phosphatase activity.

In the *AHL* overexpressor lines, wild type *PHT1-4*, *Pho1H1* and At1g73010 phosphatase transcript levels were re-established (Fig. S3), further confirming the complementation of the *fry1* phenotype by AHL activity. As expected, the overexpression of *AHL* was able to complement the *PHT1;4:GUS* induction in *fry1* (data not shown). These results strongly suggest that the lack of only the 3′,(2′),5′-bisphosphate nucleotide phosphatase activity is responsible for all the phenotypes analyzed in the current study.

Interestingly, Kim and von Arnim [4] showed that the *35S:AHL* construct complements the aerial phenotypes of *fry1-6*. *In vivo* analysis of PAP and IP3 levels in Col and *fry1* mutants (Estavillo and Pogson, personal communication) confirm our conclusion that only the lack of the 3′ (2′),5′-bisphosphate nucleotide phosphatase activity of FRY1, and the concomitant PAP accumulation, are responsible for all *fry1* mutant phenotypes described here.

### In roots, the FRY1-GFP fusion protein is mainly located in the inner mature tissues and in meristems

**Figure 5:**
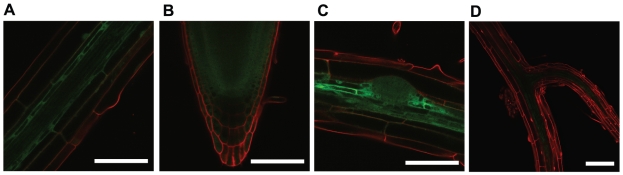
Pattern of expression of the FRY1-GFP fusion protein in roots. Roots of a *fry1-7* mutant complemented with a *pFRY1*:*FRY1:GFP* construct were observed by confocal microscopy. (A) Mature root. (B) PR meristem. (C) LR primordium. (D) Emerged LR. Scale bars are 75 µm in A, B and C, and 150 µm in D.

The *PHT1;4:GUS* expression in the internal cell layers of *fry1* roots (Fig. 1B) suggests that *FRY1* may be expressed in these tissues. To verify this hypothesis, we transformed the *PHT1;4:GUS/fry1-7* mutant line with a GFP-tagged FRY1 genomic construct (*pFRY1*:*FRY1-GFP*). This construct is functional because it complemented the root development defects of *fry1-7* (data not shown). In the mature part of the roots, the FRY1-GFP fluorescence was detected in all cell layers, except the epidermis (Fig. 5A), with strongly enhanced expression in the pericycle and stele regions of the mature part of the PR. The fusion protein was strongly expressed in the PR meristem and the root cap (Fig. 5B). It was also detected in the LR primordia (Fig. 5C), emerged LR (Fig. 5D) and LR meristems (data not shown). Therefore, the overall *FRY1* expression pattern largely overlaps with the *PHT1;4:GUS* expression pattern observed in a *fry1-7* mutant background (Fig. 1B). This suggests that the role of FRY1 on *PHT1;4* expression is tissue-specific, as the induction appears limited to the regions where *FRY1* shows the highest expression level *in planta*.

### Grafting experiments reveal two modes of action for FRY1

**Figure 6:**
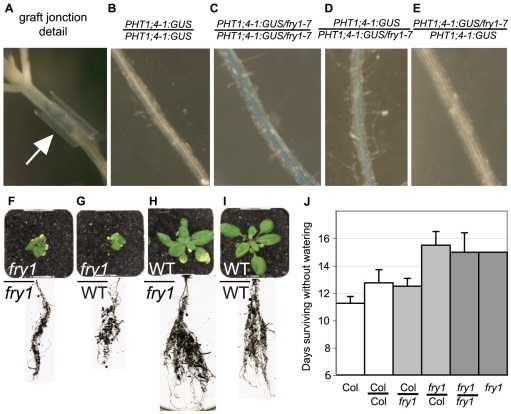
FRY1 in shoot complements the root growth defects of *fry1* but not the expression of the *PHT1;4:GUS* reporter gene. (A) The grafting junction. Arrow indicates the silicon ring. (B–E) The different graft combinations (scion/root) between the *PHT1;4:GUS* line and the *PHT1;4:GUS* line are indicated. Below are the corresponding pictures of a grafted root after the overnight GUS staining. (F–I) Shoot and root phenotypes of the different graft combinations (scion/root) between the wild type and the *fry1* mutant, after 4 weeks of growth in soil. Note that the root growth of *fry1* is complemented by the wild type shoot (H), but wild type roots display a *fry1* phenotype when grafted with a *fry1* scion (G). (J) Survival rate after withholding watering of plants with different grafting combinations show that the drought tolerance phenotype of *fry1* is determined by the scion genotype. Data are from one representative experiment out of three. Error bars represent standard error.

The expression of *FRY1* and *PHT1;4* in the root stele led us to examine whether the *PHT1;4:GUS* induction in *fry1* could be complemented by a mobile component moving from the shoot. We took advantage of the *PHT1;4:GUS* reporter in our *fry1-7* allele to examine whether FRY1 acts in a tissue-autonomous way. Micrografting experiments were set up with *in vitro* plantlets (Fig. 6A), using the parental line (*PHT1;4:GUS*) and the mutant line (*PHT1;4:GUS/fry1-7*). As expected in high Pi media, we did not observe any GUS expression in roots of the control *PHT1;4:GUS//PHT1;4:GUS* grafts (Fig. 6B), whereas those of the control *PHT1;4:GUS/fry1-7*//*PHT1;4:GUS/fry1-7* grafts showed strong GUS staining in the central cylinder and the pericycle (Fig. 6C). Grafting a *PHT1;4:GUS* scion on a *PHT1;4:GUS/fry1-7* root stock (Fig. 6D) generated roots with the *PHT1;4:GUS/fry1-7* GUS expression pattern, whereas grafting of a *PHT1;4:GUS/fry1-7* scion on a *PHT1;4:GUS* root stock resulted in roots with the GUS pattern of *PHT1;4:GUS* plants (Fig. 6E). Therefore, a wild type *FRY1* in the shoot does not complement the mutant expression pattern of *PHT1;4:GUS* in the *fry1-7* root stock.

Then, we tested whether a wild type shoot could complement the root growth phenotype of *fry1* (Fig. 6F–I). Five weeks after grafting, we observed that wild type roots remained small like *fry1* roots (Fig. 6G). Conversely, the *fry1* roots grew like wild type when grafted on a wild type shoot (Fig. 6H). These grafting experiments indicate that the root growth defect of the *fry1* mutant is complemented by the wild type shoot. We can hypothesize that a mobile component produced only in leaves is necessary in the root pericyle to exhibit normal root growth. When the aerial part of a graft is unable to synthesize this mobile component (*fry1* scion), the roots are less sensitive to auxin and therefore initiate less LR.

**Figure 7:**
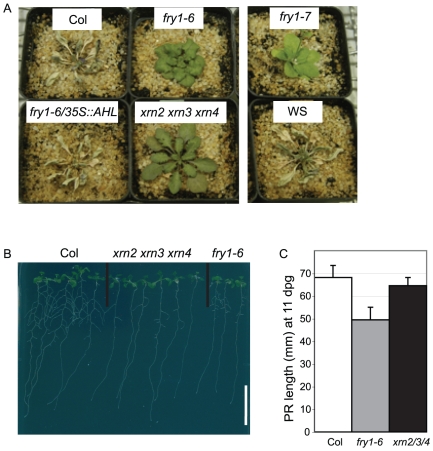
The *xrn2 xrn3 xrn4* triple mutant mimics *fry1* drought tolerance and root architecture phenotypes. (A) Dehydration experiment on 4 week-old soil grown plants of the indicated genotypes. Two independent experiments, with 6 to 17 plants per genotype per experiment, gave the same results. (B) Root architecture phenotype of the *xrn2 xrn3 xrn4* triple mutant compared to the wild type Col and the *fry1-6* mutant at 11 dpg. Scale bar is 20 mm. (C) Primary root length of the same plantlets, at the same age. Note that the length of Col and *xrn2 xrn3 xrn4* primary roots are not significantly different.

To help in the interpretation of these contrasting results, we investigated whether grafting could also restore other known characteristics of *fry1* mutants. Wilson *et al*. [2] have shown that *fry1* mutants tolerate drought stress up to 50% longer than wild type controls. We used our different graft combinations to test whether this tolerance depends on the root system or on the shoot. Fig. 6J shows than when a wild type scion is grafted on a *fry1* root it is just as tolerant to drought as when it is grafted on a wild type root (p>0.1). In contrast, wild-type root-stocks did not adversely affect the tolerance of *fry1* scions compared to their endogenous roots (p>0.1). By day 12, whatever the grafting combination, most of the wild type scion plants were dead whereas the *fry1* scions survided an additional 3 days on average (p>0.1). These experiments demonstrate that the root genotype does not determine the drought tolerance of the aerial part of the plant, indicating that the lack of *FRY1* in the leaves is sufficient for drought tolerance. We therefore investigated whether the drought tolerance phenotype of *fry1* was due to the lack of FRY1 3′ (2′),5′-bisphosphate nucleotide phosphatase activity. The *fry1-6/35S*::*AHL* overexpression line displayed a wild type level of drought tolerance (Fig. 7A), indicating that the drought tolerance of *fry1* is due to the lack of 3′ (2′),5′-bisphosphate nucleotide phosphatase activity.

The complementation of the *fry1* root development phenotype and drought resistance by a wild type scion and the non-complementation of the *PHT1;4:GUS* induction by the wild type scion indicates that FRY1 regulates different aspects of plant physiology by two different mechanisms. Presumably, a mobile component produced by leaves expressing *FRY1* is moving to roots and regulating root development but not *PHT1* expression.

### The *xrn2 xrn3 xrn4* triple mutant displays the *fry1* lateral root and drought tolerance phenotypes but does not affect primary root

It has been proposed that XRN activity is inhibited in a *fry1* background [9], likely because of the accumulation of the XRNs inhibitor PAP (Estavillo and Pogson, personal communication). Accordingly, both *fry1* and the *xrn* mutants accumulate RNA intermediates of miRNA-directed post-transcriptional regulation and share common traits [9]. To further analyze the role of XRN in the *fry1* phenotype, we generated an *xrn2 xrn3 xrn4* triple mutant that was fertile, unlike the sterile *xrn2 xrn3* double mutant. Thus the triple *xrn2 xrn3 xrn4* mutant facilitated *in vitro* root analysis without antibiotic selection, which has negative consequences on root development. Although the mechanism for the partial phenotypic rescue is unclear, it suggests that *xrn4* mutations act to partially suppress the *xrn2 xrn3* phenotypic effects. We found that the lateral root phenotype of the *xrn2 xrn3 xrn4* triple mutant was similar to that of *fry1* (Fig. 7B), whereas the primary root of the triple mutant was not significantly reduced compared to wild type (Fig. 7C). We also found that the *xrn2 xrn3 xrn4* triple mutant tolerated a drought stress like the *fry1* mutants (Fig. 7A). Altogether, these results suggest that the pleiotropic phenotype of the *fry1* mutants results, at least in part, from a general perturbation in XRN activities.

### The *PHT1;4:GUS* induction in *fry1* is unrelated to its action on XRNs

We investigated whether the *xrn* mutations could mimic the induction of *PHT1;4:GUS* observed in *fry1*. For this, we crossed the *PHT1;4:GUS* parental line to the different single, double and triple *xrn* mutant lines. We confirmed the crosses by checking that the GUS marker was active in ¾ of the F2 when plants were grown in phosphate deficient media (Table 1). Interestingly, in plantlets grown in complete media, we never observed GUS-stained roots (Table 1) demonstrating that although the *xrn* mutations can mimic many of the *fry1* mutant phenotypes (root architecture, leaf shape, drought tolerance), they do not mimic the induction of the *PHT1;4* locus. In addition, a qRT-PCR analysis of the *xrn2 xrn3 xrn4* triple mutant confirmed that the XRN activities are not responsible for the up-regulation of *PHT1* genes (Fig. S4). Indeed, the assayed mutants (*xrn4-6* and the *xrn2 xrn3 xrn4*) showed the same level of *PHT1;4*, *PHT1;7*, *Pho1H1* and AT1g73019 transcripts as the Col control. Thus, this analysis further confirmed that the *xrn* mutations do not mimic the induction of the *PHT1;4* locus, nor the general induction of phosphate-starvation genes observed in the *fry1* background.

**Table 1:** *PHT1;4:GUS* expression in different *xrn* backgrounds.

	Number of F2 plantlets stained/total nb of plantlets assayed	
Genetic cross	on Pi depleted media	on Pi complete media
*xrn2* X *PHT1;4:GUS*	16/24	0/24
*xrn3* X *PHT1;4:GUS*	17/24	0/24
*xrn4* X *PHT1;4:GUS*	34/45	0/204
*xrn2 xrn4* X *PHT1;4:GUS*	39/58	0/474
*xrn2 xrn3 xrn4* X *PHT1;4:GUS*	18/22	0/347

The F2 progeny of the indicated crosses were grown 7 to 10 days on either a complete or depleted Pi media before the GUS staining. Results on the Pi depleted media serve as a positive control for the presence of the *PHT1;4:GUS* transgene. Note that on a Pi-rich media, none of the seedlings expressed the *GUS* reporter gene.

**Figure 8:**
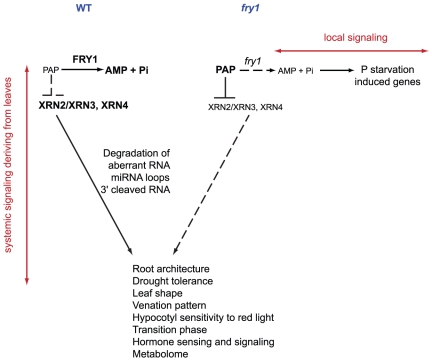
Model accounting for the dual mode of action of FRY1. The expression of *FRY1* in the shoot is essential for root growth, drought resistance, and likely many other developmental aspects. This systemic mode of action relies on the XRN activities. By contrast, FRY1 has a local (i.e. not systemic) effect on the expression of the *PHT1* genes and other phosphate starvation markers in roots; this effect depends on Pi accumulation but not on the XRN activities.

The inability of *xrn* mutants to induce *PHT1;4* transcription argues in favor of a model whereby FRY1 has two physiological roles for the 3′,(2′),5′-bisphosphate nucleotide phosphatase activity (Modeled in Fig. 8). On one hand, the PAP accumulation in the mutant represses XRN activity, altering various phenotypes linked to the deregulation of the silencing machinery (root architecture, drought tolerance, leaf shape, hypocotyl sensitivity to red light, hormonal sensing and signaling). Indeed, *fry1* late flowering, short petioles and hypocotyl hypersensitivity to red light phenotypes are largely mimicked by the *xrn2 xrn3* double mutant [4]. The root architecture of the *xrn4* single mutant has been described as being similar to that of *fry1* [5]. However, only the *xrn2 xrn3 xrn4* triple mutant presents *fry1-*like lateral root architecture defects in our conditions (Figs. 7B, C). The *xrn4* mutant presents wild type LR development (Fig. S5A) and a PR length intermediary between the Col and the *fry1-6* PR lengths (Fig. S5B). In this mutant, the levels of the phosphate-starvation markers that appear induced in f*ry1* are comparable to the control levels (Fig. S4). In addition, the *xrn4* single mutant is not drought tolerant (Estavillo and Pogson, personal communication). Interestingly, the *xrn2 xrn3* drought tolerance level is intermediary between the wild type and the *fry1* drought tolerance levels (Estavillo and Pogson, personal communication), whereas the rosette phenotype of the double mutant is similar to that of the *fry1-6* mutant [9]. Moreover, both *fry1* mutants and the *xrn2 xrn3 xrn4* triple mutant tolerate a drought stress that is lethal for the wild type controls (Fig. 7A), even though the rosette shape of the triple mutant is quite different from the *fry1* rosette (compare the petiole length in *fry1-6* and the *xrn2 xrn3 xrn4* triple mutant in Fig. 7A). Thus, the drought tolerance observed in both *fry1* and *xrn2 xrn3 xrn4* triple mutants is not linked to a reduced leaf biomass and transpiration, but rather to reduced XRN activity. All of the phenotypes linked to perturbations in XRN activities can be complemented by grafting, suggesting the presence of a systemic signal (Fig. 8, left). On the contrary, the induction of phosphate starvation markers is likely linked to a local effect of *FRY1* expression (Fig 8, right). It is not complemented by a wild type scion and is not mimicked by the *xrn* mutations or linked to the root architecture phenotype.

We propose that this effect could be a result of the by-products of FRY1 activity, more specifically the result of the conversion of PAP into AMP + Pi. A reduction of FRY1 activity likely leads to a slight reduction of AMP and phosphate levels (along with an accumulation of PAP) in the tissues where FRY1 is normally very active (the root pericycle, central cylinder and meristems). The reduction of Pi availability would lead to the transcriptional induction of several phosphate starvation genes (including the *PHT1;4:GUS* marker) in these cell layers. This effect is not complemented by a wild type scion and is not mimicked by the *xrn* mutations or linked to the root architecture phenotype.

### Conclusion

Long-distance signaling is used by plants to coordinate shoot and root development. Despite the importance of such coordination, only a few genes have been shown to regulate root development in a systemic way. For example, in *Lotus japonicus*, the use of reciprocal and self-grafting studies with the hypernodulating mutant *har1* have shown that the shoot genotype is responsible for the negative regulation of nodule development. Therefore, HAR1 in shoots mediates systemic regulation of nodulation [16]. There are also few examples of root genes regulating shoot development by long-distance signaling. For example, the *BYPASS1* locus is required in Arabidopsis to prevent constitutive production of a root-derived graft-transmissible signal that is sufficient to inhibit leaf initiation, leaf expansion and shoot apical meristem activity [17]. We demonstrate here that *FRY1* in shoots controls root development in Arabidopsis.

We have identified a novel FRY1 function modulating the transcription of several Pi starvation markers in the root stele. This is the first *fry1* mutant phenotype reported to be independent of XRN activities. Instead, it is likely depending on FRY1 impact on the root cytosolic Pi pool, in stele and pericycle cell layers. Interestingly, this phenotype is not complemented by a wild type scion and therefore acts locally.

## Materials and Methods

### Plant material and growth conditions

The *PHT1;4:GUS* line (originally referred to as *pht1;4-1* in [10]) was isolated from a T-DNA mutagenized *A. thaliana* ecotype *Wassilewskija* (Ws) seeds collection, obtained from INRA [18]. *fry1-6* (SALK_020882), *xrn2-1* (SALK_041148), *xrn3-3* (SAIL_1172C07) and *xrn4-6* (SALK_014209) mutants as well as the *xrn2 xrn3*, *xrn2 xrn4* and *xrn3 xrn4* double mutants have been described before [9]. Because *XRN3* and *XRN4* are genetically linked on chromosome 1, whereas *XRN2* is on chromosome 5, the *xrn2 xrn3 xrn4* triple mutant was generated by crossing *xrn2 xrn4* to *xrn3 xrn4* so that 1/16 of the F2 plants would be homozygous for the three mutations (*xrn2* and *xrn3* being genetically independent).

For physiological analyses and RNA extractions, seeds of Col-0, Ws, *PHT1;4:GUS*, and the different *fry1* mutant alleles were cultivated as described before [10]. For drought tolerance tests, plants were grown in individual pots in short days for 4 weeks with standard watering conditions (once a day). Watering was stopped for 13 days and the pots were then rehydrated for 3 days before the observations. Alternatively, after the onset of wilting, survival of the plants was quantified by measuring chlorophyll fluorescence as described [2], [19].

### Mutagenesis, screening conditions and histology

Approximately 3000 seeds of *PHT1;4:GUS* were mutagenized with a 0.3% solution of Ethyl methane sulfonate (EMS) as described [20]. Seeds were sown and the M1 plants were cultivated to obtain the M2 generation. Around 30 seeds of each M2 line (1400 lines) were sown in 6-well Petri dishes (NUNC) containing a modified Hoagland medium (1 mM MgSO_4_, 2 mM Ca(NO_3_)_2_, 1.7 mM KNO_3_, 1.6 µM Fe, 46.2 µM H_3_BO_3_, 9.1 µM MnCl_2_, 0.87 µM ZnSO_4_, 0.32 µM CuSO_4_, 1.03 µM Na_2_MoO_4_, 0.5 mM NH_4_H_2_PO_4_). After 10 days, seedlings were screened for their GUS expression as described [10]. Histological analysis were performed as described [21].

### Genetic analysis and positional cloning of the mutant

The mutant line was backcrossed three times to the parental line (*PHT1;4:GUS*) to test the linkage of the phenotype to a single Mendelian recessive mutation. For mapping purposes, a mutant plant (Ws ecotype) was crossed with a wild-type Col plant. Linkage analysis was performed with the F2 progeny of this cross as described [22]. DNA from F2 seedlings displaying the mutant phenotypes (GUS staining of a root piece from seedlings grown on complete media) was prepared as described [23]. Single Sequence Length Polymorphism markers [24] distributed on the five chromosomes and polymorphic between the Ws and Col accessions were tested on the extracted DNA. Thermal cycling consisted of an initial denaturation at 94°C for 2 minutes, followed by 38 cycles of denaturation step at 94°C for 15 seconds, annealing at the respective Tm of each oligonucleotide pair for 20 seconds, and extension at 72°C for 45 sec. At the end of the reactions, the PCR products were allowed to extend for 2 minutes at 72°C.

To identify the mutant locus on chromosome 5, the Gramene Simple Sequence Repeat Identification Tool (SSRIT, http://www.gramene.org/db/markers/ssrtool) was used to generate new markers in the area surrounding *FRY1*.

### Mutant complementation and tissue localization of FRY1

The *FRY1* genomic fragment (1960 bp) and an additional 753 bp upstream region was PCR cloned by standard molecular techniques in the Ws accession. After sequencing in the pENTR/D-TOPO (Invitrogen, Carlsbad, USA), an LR clonase (Invitrogen, Carlsbad, USA) reaction was used to clone the genomic fragment in the binary vector pGWB4 [25]. Then, the Arabidopsis *fry1-7* mutant was transformed by a simplified floral dip method [26]. Similar construct were built with the *FRY1* cDNA (1221 bp) with or without a C-terminal GFP fusion, under the control of the 35S promoter. Primary transformants were selected in medium containing 50 µg/L hygromycin. Their progeny was screened for root phenotype and GFP expression in standard *in vitro* growing conditions using a Leica SP2 AOBS inverted confocal microscope (Leica Microsystems, Germany) equipped with an Argon ion laser. Prior to confocal observation, plantlets were stained 3 min in 100 µg/mL propidium iodide (PI). Leaf shape, flowering time and GFP expression in mature plants were screened in soil-grown plants, both in short and long days conditions.

### Analysis of root architecture

Seedlings were photographed at different times after germination and PR and LR length were measured with the ImageJ software (http://rsb.info.nih.gov/ij/). To determine the speed of growth of the main root, photographs were taken at 8, 11 and 14 days post germination (dpg) from which PR length was measured. The daily growth was calculated accordingly. To measure single cell length above the differentiation zone, roots were briefly stained with ruthenium red and observed with a bright field microscope (Leica DMRXA, 20x objective). At least 30 cells for each of 12 different roots per genotype were measured using a micrometric lens. To estimate the size of the proximal meristem (PM), the number of undifferentiated cells in the cortex was measured in at least 30 roots per genotype as described [27], [28]. PI-stained roots (3 min in 100 µM PI) were observed by confocal laser scanning microscopy. PI was excited at 514 nm and imaged using a custom 610–720 nm band pass emission filter. To determine the number of LR primordia at different stages of development, we used a Nomarsky optical microscope, as described [29]. All experiments were performed at least three times.

### Grafting experiments

Grafting was performed according to [30]. Parental and mutant lines were sown *in vitro* on a MS/10 medium. Four days after sowing they were cut at the hypocotyl level to separate the aerial and root parts. A 0.3 mm diameter silicon ring (Silastic Laboratory tubing, Dow Corning, USA) was used to maintain the aerial seedling scion and the rootstock together to allow fusion. After five days, successful grafts were transferred to fresh medium for 48 hours, followed by GUS staining for 16 h at 37°C. Alternatively, established grafts were put on soil, either on large soil-filled plates or in pots and grown in the greenhouse for 4 weeks in order to assess the root architecture and the drought tolerance of the grafts. For drought tolerance, plants were either cultured in long days (12 h light, 12 h dark), before watering was withheld, then survival of the plants was determined as described [19] or cultured in short days (8 h light, 14 h dark) using a mix of ¼ soil and ¾ sand and an immersion watering per day. Phenotype was assessed after 13 days without watering followed by three days were watering of the individual pots was resumed.

### Molecular and gene expression analysis

For gene expression analysis, total RNA was extracted from rosettes and roots of 10 day-old plantlets of the *PHT1;4:GUS* parental line and the *PHT1;4:GUS*/*fry1-7* mutant, grown in MS/10 medium as described previously [12]. cRNA was prepared using the manufacturer's instructions (www.affymetrix.com support technical manual expression_manual.affx). Labeling and hybridization on the ATH1 microarray and data analysis were performed according to [12]. Microarray data has been deposited at the EMBL database with the accession number E-MEXP-2483 (www.ebi.ac.uk/arrayexpress) and was used in the present work to identify candidate genes during the positional cloning of the mutant locus.

To analyze mRNA splice variants, 10 µg of total RNA from roots and leaves were treated with DNase1 (Roche Diagnostics, Meylan, France) for 15 min at 37°C and were used for the reverse transcription reaction using the AMV Reverse Transcriptase (Roche Diagnostics, Meylan, France) according to the manufacturer's instructions. Specific primers (sequence available on request) were used to amplify *FRY1* transcripts, both in the wild type and the mutant backgrounds. DNA cloning and sequencing were performed by standard procedures [31].

RTqPCR analyses were performed after reverse transcription (kit from GE Healthcare) and amplification (Applied ABI7000). Primer efficiency factors were measured for each gene and GapC and ROC3 were used as reference genes. Primer sequences are available upon request.

### Production of Arabidopsis transformants expressing *pAT1G73010::LUC*

A DNA fragment corresponding to 2001 bp of the promoter driving the expression of the AT1G73010 gene (ending right before the ATG) was PCR amplified and cloned into the pENTER-D-TOPO vector. The fragment was recombined into the pBGWL7 vector [32] using LR clonase. After sequencing confirmation, the vector was introduced into C58C1 *Agrobacterium tumefaciens* cells. Arabidopsis plants were transformed using a modified floral dip method [26], and transformed plants were selected using Basta (T1).

Bioluminescence detection was performed on the T2 generation (8 day-old plantlets) using a UPLSAPO 4X dry objective (N.A. 0.16) or a LUCPLFLN 40X dry objective (N.A. 0.6) mounted on an Olympus LV200 Luminoview microscope coupled to an ANDOR iKon-M DU934 camera. Images were acquired with an exposure time of 2 min (4X objective) or 4 min (40X objective). Contrast and brightness of the images were adjusted in ImageJ.

## Supporting Information

Figure S1 **Mutant complementation assays and leaf phenotype of *PHT1;4:GUS*/*fry1-7* mutant.** (A) Complementation of the *fry1-7* mutant. The progeny of a plant heterozygous for a T-DNA carrying a *35S::FRY1* cDNA construct is shown. Asterisks indicate non-complemented *fry1-7* mutant plantlets that, presumably, did not inherit the transgene. (B) Picture of the rosette of the 3 week-old *PHT1;4:GUS* line (left) and the *PHT1;4:GUS*/*fry1-7* mutant (right) grown on soil under short day conditions. (C) 6 week-old *PHT1;4:GUS* line (left) and the *PHT1;4:GUS*/*fry1-7* mutant (right) grown in long day conditions. (D) Complementation of the *fry1-6* mutant with a *35S::AHL* cDNA. The Col control (left), the *fry1-6* mutant (middle) and the complemented line (*fry1-6*/*35S::AHL*) (right) are shown. White scale bars are 20 mm.
(EPS)

Figure S2 **The *pAT1G73010::LUC* construct reveals the stele specificity of gene expression in Arabidopsis roots and the phosphate starvation induction.** (A) Transmitted light image and (B) bioluminescence signal of *pAT1G73010::LUC* plantlets grown for 4 days on P depleted medium then for 4 days on complete medium (plantlet on the left) or for 8 days on P depleted medium (plantlet on the right). Scale bar, 1 mm. (C) Close up of a mature part of a root from a plantlet grown for 8 days on P depleted medium (overlay of transmitted light and bioluminescence signal). The bioluminescence signal is only detected in the central cylinder. Scale bar, 100 µM.
(EPS)

Figure S3 **Expression of phosphate-starvation induced genes in the *35S:AHL* complemented line.** (A) Quantitative real time PCR of the *PHT1;4* transcripts in Col, *fry1-6* and *fry1-6/35S::AHL* roots. (B) Quantitative real time PCR on the *Pho1H1* transcript in Col, *fry1-6* and *fry1-6/35S::AHL* roots. (C) Quantitative real time PCR on the *At1g73010* phosphatase transcript in Col, *fry1-6* and *fry1-6/35S::AHL* roots. Biological triplicates were performed and all samples were analyzed with technical triplicates. White bars correspond to Col roots, grey bars to *fry1-6* roots and black bars to *fry1-6/35S::AHL* roots. Standard deviations are shown.
(EPS)

Figure S4 **Expression of phosphate-starvation induced genes in the *fry1-6*, *xrn4* and *xrn2 xrn3 xrn4* mutants.** Quantitative real time PCR of the *PHT1;4* transcript (A), the *PHT1*,*7* transcript (B), the *Pho1H1* transcript (C) and the *At1g34010* phosphatase transcript (D) in Col, *fry1-6*, *xrn4-6* and *xrn2 xrn3 xrn4* roots. Biological triplicates were performed and all samples were analyzed with technical triplicates. White bars correspond to Col, pale grey to *fry1-6*, dark grey to *xrn4-6* and black bars to *xrn2 xrn3 xrn4* mutant. Standard deviations are shown.
(EPS)

Figure S5 **Root development and primary root length of the *xrn4-6* mutant.** (A) The general *in vitro* development of Col, *xrn4-6* and *fry1-6* mutants 11 dpg. Scale bars are 20 mm. (B) PR length was measured at 11 dpg. White bars correspond to Col, grey bars to *fry1-6* and black bars to *xrn4-6* mutant. Standard deviations are shown.
(EPS)
